# Filtered reproductive long non-coding RNAs by genome-wide analyses of goat ovary at different estrus periods

**DOI:** 10.1186/s12864-018-5268-7

**Published:** 2018-12-04

**Authors:** Yong Liu, Bing Qi, Juan Xie, Xiaoqing Wu, Yinghui Ling, Xinyan Cao, Feng Kong, Jing Xin, Xin Jiang, Qiaoqin Wu, Wenying Wang, Qingmei Li, Shengnan Zhang, Fengrui Wu, Di Zhang, Rong Wang, Xiaorong Zhang, Wenyong Li

**Affiliations:** 1 Key Laboratory of Embryo Development, Reproductive Regulation of Anhui Province, Fuyang Normal University, Fuyang, 236041 Anhui China; 2 School of Life Sciences, Taishan Medical University, Taian, 271016 Shandong China; 3 Anhui Provincial Laboratory of Animal Genetic Resources Protection and Breeding, College of Animal Science and Technology, Anhui Agricultural University, 130 Changjiang West Road, Hefei, 230036 Anhui China; 4 Institute of Special Animal and Plant Sciences, Chinese Academy of Agricultural Sciences, No.4899 Juye Street, Jingyue District, Changchun, 130112 China

**Keywords:** LncRNA, Genome-wide, Luteal phase, Follicular phase, Goat ovary

## Abstract

**Background:**

The goat is an important farm animal. Reproduction is an important process of goat farming. The ovary is the most important reproductive organ for goats. In recent years, an increasing number of long non-coding RNAs (lncRNAs) have been implicated in the regulation of mammal reproduction. However, there are few studies on the function of lncRNAs in reproduction, particularly lncRNAs in the ovary.

**Results:**

The sequencing of goat ovaries generated 1,122,014,112 clean reads, and 4926 lncRNAs and 1454 TUCPs (transcripts of uncertain coding potential) were identified for further analysis by using the coding potential analysis software, CNCI, CPC and Pfam-sca. There were 115 /22 differential lncRNAs /TUCPs transcripts between the ovaries of the luteal phase and the follicular phase. We predicted the related genes of lncRNA /TUCP based on co-expression and co-localization methods. In total, 2584 /904 genes were predicted by co-expression, and 326/73 genes were predicted by co-localization. The functions of these genes were further analyzed with GO and KEGG analysis. The results showed that lncRNAs /TUCPs, which are highly expressed in goat ovaries in the luteal phase, are mainly associated with the synthesis of progesterone, and we filtered the lncRNAs /TUCPs, such as XR_001918177.1 and TUCP_001362, which may regulate the synthesis of progesterone; lncRNAs /TUCPs, which are highly expressed in goat ovaries in the follicular phase, are mainly associated with oogenesis and the maturation of oocytes, and we filtered the lncRNAs /TUCPs that may regulate the oogenesis and maturation of oocyte, such as XR_001917388.1 and TUCP_000849.

**Conclusion:**

The present study provided the genome expression profile of lncRNAs /TUCPs in goat ovaries at different estrus periods and filtered the potential lncRNAs /TUCPs associated with goat reproduction. These results are helpful to further study the molecular mechanisms of goat reproduction.

**Electronic supplementary material:**

The online version of this article (10.1186/s12864-018-5268-7) contains supplementary material, which is available to authorized users.

## Background

The goat is an important farm animal that can provide people with farm products, such as meat [1], skin, wool, milk and mutton, which are widely used in production and daily life. Goat farming has a good market prospect, and reproduction is one of the key processes in goat farming. Increasing production efficiency requires intensive research on reproductive mechanisms. The ovary is the most important reproductive organ of female goats, as the ovary can directly mediate the maturation of oocytes and secrete female hormones, which has a significant influence on the fecundity of goats [2]. There are two main functions of the goat ovary. The first function is to produce oocytes and ovulate. Subsequently, the oocyte matures in the ovary and has the potential for fertilization in the ovary. The second function is to secrete steroid hormones, such as estrogen, progesterone and androgen. This series of gonadal hormones greatly influences goat breeding, controls follicular development and maintains goat rutting [3].

During the reproductive process, the estrous cycle of goats can be divided into follicular and luteal phases. The goat ovaries in these two periods differ in the development of follicles, oogenesis and the maturation of oocytes, the formation of the corpus luteum, the maintenance of pregnancy, and the secretion of gonadal hormone. In the follicular phase, the follicles in the ovary grow, and the follicle development process is regulated by endocrine, paracrine and gene expression. Genes can feedback-regulate follicular development by expressing products, such as FOXL2, which promotes the growth, development and anti-apoptosis of follicles [4, 5]. The amount of estrogen in the ovaries increases during this period, prompting the animals to become sexually aroused, and most mammals ovulate at the end of this period. During oocyte maturation, a large number of maternal factors accumulate, and this process requires large amounts of gene expression. Ovaries in the luteal phase primarily secrete progesterone, which acts on the reproductive tract and the uterus, promoting embryo implantation and maintaining pregnancy. The formation of the corpus luteum in mammals is vital for steroid biosynthesis [6], and sex steroid hormones, such as estrogen, progesterone and testosterone, play a key role in sex differentiation, reproductive function and behavior in mammals [7].

Non-coding RNA, which was previously considered “transcriptional noise” without biological function, is particularly important. These non-coding RNAs include miRNA and lncRNA [8], most of which are lncRNAs. LncRNAs are transcription RNAs > 200 nt in length, having complex structure and lacking the ability to code protein, as they have no open reading frame; these molecules are regulated by the recognized transcription factor and show specific expression [9]. LncRNAs are widely distributed and have been identified in animals, yeast, plants and even viruses [10–14]. Xist is a lncRNA molecule that plays a key role in the inactivation of the x chromosome in mammals [15]. The discovery of Xist officially prompted lncRNA research in the field of reproduction, and a growing number of lncRNA have been implicated in the regulation of mammalian ovary development [16, 17]. Studies have shown that lncRNAs can regulate reproductive processes, such as the maturation and fertilization of oocytes and the embryonic development in the ovaries of female goats [18], and the maturation of oocytes is an important process that causes ovulation in goats [19]. Therefore, lncRNA plays an important role in regulating the growth and development of oocytes. Recent studies have shown that lncRNAs play an important role in cell development, proliferation, differentiation, apoptosis and other biological processes [20–24] as well as vital roles in lactation, ovary and embryo development, sperm maturation and so on [25]. An increasing number of lncRNAs have also been implicated in the regulation of mammalian breeding. However, there are few studies on the effects of goat lncRNAs on reproduction.

RNA-seq is a second-generation sequencing technology to analyze and identify the biological functions of lncRNAs [26]. In the present study, by using RNA-seq, we explored the genome-wide expression profiles of lncRNAs /TUCPs in goat ovaries at the follicular and luteal phases and predicted additional potential lncRNA /TUCP molecules. The differential expression of lncRNAs /TUCPs in the ovaries at the follicular and luteal phases was explored. We performed GO and KEGG enrichment analyses of the co-located and co-expressed genes of these different lncRNAs /TUCPs to investigate the role of these lncRNAs /TUCPs in goat reproduction. These findings will provide a theoretical basis for improving the reproduction of goats.

## Methods

### Ovary procurement

The Institutional Animal Care and Use Committee of Fuyang Normal University approved the present study. The ovaries in follicle and luteal stage of the Anhui white goat were selected as the animal tissues in the present study. The ovaries, which were collected from a slaughterhouse, were sent to the laboratory within 4 h. The ovaries were washed with physiological saline containing double antibodies and then were placed into an RNase-free centrifuge tube, labeled, frozen in liquid nitrogen, and stored at − 80 °C within 20 days prior to being sent to Beijing Connaught Qizhiyuan Bioinformatics Co., Ltd. for RNA sequencing.

### RNA extraction and library construction

First, total RNA was extracted from the goat ovaries at the follicular and luteal phases. Then, 1% agarose gel electrophoresis was prepared to monitor the degradation and contamination of the extracted RNA. The purity and concentration of the RNA were respectively measured by the NanoPhotometer® spectrophotometer (IMPLEN, CA, USA) and the Qubit ® 2.0 Fluorometer (Life Technologies, CA, USA). Finally, the RNA integrity was assessed by the RNA Nano 6000 assay kit (Agilent Technologies, CA, USA) with the Bioanalyzer 2100 system. To prepare the RNA sample, 3 μg of RNA per ovary was used as input material. First, the Epicenter Ribo-zero™ rRNA Removal Kit (Epicenter, USA) was used to remove ribosomal RNAs, and rRNA was removed by ethanol precipitation. Then, the NEBNext, hyperdirectional RNA library preparation kit (NIB, USA) was used to generate the sequencing library by using rRNA-depleted RNA according to the manufacturer’s instructions. Subsequently, sequencing libraries were generated by using rRNA-depleted RNA by the NEBNext® Ultra™ Directional RNA Library Prep Kit for Illumina® (NEB, USA) according to the manufacturer’s instructions.

### Sequencing and quality control

TruSeq PE Cluster Kit v3-cBot-HS (Illumina) was used to cluster the cBot clusters. Then, the library was sequenced on Illumina Hiseq 4000, and 150 bp paired end readings were produced. The raw reads in fastq format were first processed through internal Perl scripts. In this step, clean reads were obtained by removing the sequence containing the adapter, including the sequence of ploy-N and the low-quality sequence. In addition, the Q20, Q30 and GC content of the sequences were calculated. All downstream analyses are based on high-quality filtered sequences. All bioinformatics analyses were based on high-quality clean reads.

### Transcriptome assembly and encoding potential analysis

The gene model annotation files and reference genomes were directly downloaded from the genome website. Bowtie2 v2.2.8 was used to construct the index of the reference genome, and HISAT2 [27] v2.0.4 was used to compare the purified reading of the paired ends to the reference genome. Based on a reference method, the mapped reads of each sample were assembled by StringTie (v1.3.1) [28]. The novel network process algorithms and optional reassembly steps were performed by StringTie to assemble and quantitate the full-length transcript of multiple splicing variants of each locus.

Based on the structural characteristics and non-coding function of lncRNA, we set up a series of strict screening conditions to obtain high-quality lncRNA through the following five-step screen: (1) Transcripts with low expression levels, low credible single exon transcripts, and exon numbers < 2 were filtered out; (2) transcripts < 200 bp in length were filtered out; (3) through Cuffcompare software, the duplicated transcripts of the exon region of the database annotation were removed, and the lncRNA overlapped with the exon region of the splicing transcript; (4) the expression of each transcript was calculated by Cuffquant, and the transcript of FPKM< 0.5 was filtered out; and (5) finally, we determined whether the encoding potential was the key condition to determine lncRNAs. We used three coding potential analysis programs (CNCI, CPC, and Pfam-sca) to screen the transcripts, and transcripts with coding potential predicted by one or all of the three tools mentioned above were filtered out. Transcripts without coding potential were considered candidate lncRNAs. Transcripts of uncertain coding potential (TUCPs) is an additional set of transcripts. They have high evolutionary conservation but may include short open reading frames. They have potential to encode proteins and may serve either as lincRNAs or as small peptides.

### Quantification of gene expression level and differential expression analysis

The FPKMs [29] of lncRNAs and coding genes in each sample were calculated through Cuffdiff (v2.1.1). The FPKM (the number of fragments per million fragments from a single gene) was used to estimate the expression levels of lncRNA and TUCP. According to a model in the negative binomial distribution, Cuffdiff was used to determine the differential expression of the digital transcription or the gene expression data [29]. Transcripts with *p* < 0.05 were considered differentially expressed.

### The prediction of co-located and co-expressed genes

In the present study, we predicted the co-expressed and co-located genes of lncRNA /TUCP to explore the functions of lncRNAs /TUCPs. We searched the genes 100 kb downstream and upstream of lncRNAs /TUCPs and analyzed their functions. Trans-acting refers to the co-expression relationship between lncRNA and mRNA. To explore the trans roles of these molecules, we used a custom script to calculate Pearson’s coefficient between the lncRNAs and coding genes.

### GO and KEGG analysis

The GO enrichment analysis [30] of the related genes of differentially expressed lncRNAs or TUCPS was performed by the GOseq R package. GO terms *P* < 0.05 were considered significantly enriched by differentially expressed genes. KEGG [31] was used to understand the advanced features of biological systems and the utility of the database resources (http://www.genome.jp/kegg/). We examined the statistical enrichment of the related genes of differentially expressed lncRNAs or TUCPS in the KEGG pathway by using KOBAS [32] software.

## Results

### The genomic characteristics of lncRNA and TUCP in the goat ovaries

**Fig. 1:**
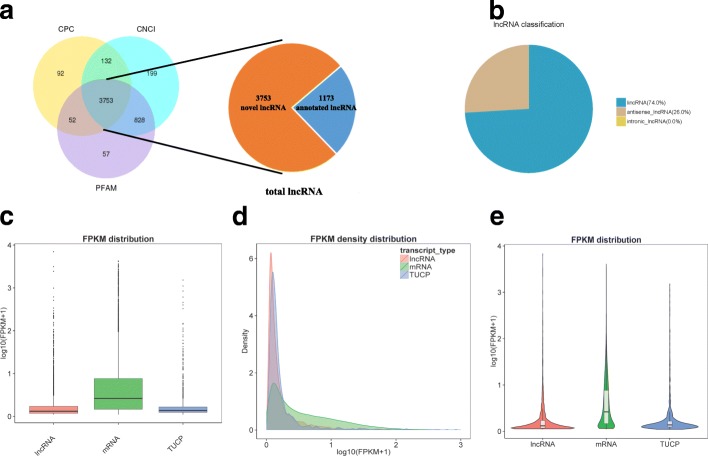
Genomic characteristics of lncRNA and TUCP in follicular and luteal goat ovaries. **a** Screening of the candidate lncRNAs in goat ovaries. **b** The classification of candidate lncRNA. **c** The boxplot of expression level (showed in log10 (FPKM+ 1)) for lncRNA and TUCP. **d** The FPKM density distribution of lncRNA and TUCP. **e** A violin plot of expression level (showed in log10 (FPKM+ 1)) for lncRNA and mRNA transcripts

In the present study, we collected five typical follicular phase and five typical luteal phase goat ovaries. The RNA sequencing was performed on the Illumina HiSeq 2500 platform, and then, we detected the expression of lncRNA and TUCP in ovarian tissue samples. Pearson’s correlation among the samples was between 0.861 and 0.935. In total, 1,164,144,548 raw reads were obtained. After filtering out the adaptor sequences, empty sequence, and low-quality sequences, 1,122,014,112 clean reads were obtained. Subsequently, a biological information analysis was conducted on the basis of the clean reads. Clean reads obtained in each library accounted for 95.31–98.03% of the raw reads. A total of 4926 lncRNAs and 1454 TUCPs were identified from the goat ovaries. Among these lncRNAs, 1173 molecules were annotated as lncRNAs and 3753 molecules were novel lncRNAs (Fig. 1a). Notably, 74% of the novel lncRNAs were lincRNAs and 26% of the novel lncRNAs were antisense lncRNAs (Fig. 1b).

The expression levels in the ovaries in follicular and luteal phases were similar, and the expression level of lncRNA and TUCP was lower than that of mRNA (Fig. 1c/d/e). The study also showed that most lncRNAs and TUCPs had fewer exons than did mRNAs: the average number of exons in the lncRNAs and TUCPs was approximately 2, while that in mRNAs was 12. Unlike mRNA, the average length of lncRNA was 805.466 nt, which was shorter than the average length of the mRNA (3477.172 nt). The length of open reading frame of mRNA was longer than that of lncRNA and TCUP. The average length of the lncRNA was 103 nt, and the average length of the TUCP was 199 nt, while the average length of mRNA was 670 nt. (Additional file 1: Figure S1A and B).

### The differences in lncRNA/TUCP expression in goat ovaries between the different estrous stages

**Fig. 2:**
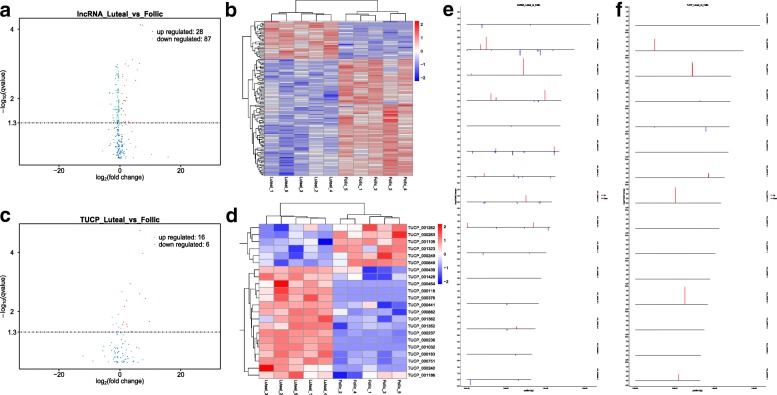
The overall differences of lncRNA and TUCP. **a**/**d**. Volcano plots of differentially expressed lncRNA/TUCP. X-axis is foldchange (log2), Y-axis is qValue(−log10). Red points indicate the up-regulated lncRNAs/TUCPs (X axis > 0); green points indicate the down-regulated lncRNAs/TUCPs (X axis < 0). **b/c** Hierarchical clustering analysis of differential expressed lncRNA/TUCP from 10 libraries. Red shows higher expression; Blue shows relatively low expression. **e/f** chromosome Distribution of differentially expressed lncRNA/TUCP. X-axis is the relative position of the gene on the chromosome and Y-axis is foldchange (log2). Red: up-regulated transcripts; green: down-regulated transcripts

In the present study, 115 lncRNAs were differentially expressed in goat ovaries for the luteal phase vs. the follicle phase, of which 28 lncRNAs were upregulated and 87 lncRNAs were downregulated (Fig. 2a). In total, 22 TUCPs were differentially expressed in the goat ovaries, of which 16 lncRNAs were upregulated and 6 were downregulated (Fig. 2c).

Systematic hierarchical clustering analysis was used to analyze the expression patterns of differentially expressed lncRNAs under different experimental conditions. Hierarchical cluster analysis was performed on all goat ovarian tissue samples (Fig. 2b). According to the heat map analysis, the expression of the same gene in the same group is almost the same, indicating that there is minimal difference between the samples in the same group; analysis of the sample cluster showed that the five samples of the luteal phase are clustered together, and the five samples of the follicular phase are clustered together, which indicates large differences between the samples in the different groups. Analysis of the gene cluster shows that the differential lncRNAs are divided into two major categories. There are 28 lncRNAs with high expression in the luteal stage and 87 lncRNAs with high expression in the follicular phase (Fig. 2b).

Systematic hierarchical clustering analysis was used to analyze the expression patterns of differential TUCPs under different experimental conditions. Hierarchical cluster analysis was performed on all goat ovarian tissue samples (Fig. 2d). The heat map analysis showed that the expression of the same gene in the same group is basically the same, indicating that there is little difference between the samples in the same group; analysis of the sample cluster revealed five samples of the luteal phase that are clustered together. The five samples of the follicular phase are clustered together, indicating large differences between the samples in different groups. Analysis of gene cluster shows that the differential TUCPs are divided into two major categories. Among them, 16 TUCPs show high expression in the luteal stage, and 6 TUCPs show high expression in the follicular phase (Fig. 2d).

To study the relationship between chromosome length and the total number of total mapped reads, we calculated the density of total mapped reads against each chromosome (the positive and negative strands) in the genome. In general, the longer whole chromosome length is, the more localized the chromosome is, and the total number of reads will increase. As shown in Fig. 2e, the differential lncRNAs are mainly concentrated on chromosomes NC-030809.1, NC-030810.1, NC-030813.1, NC-030815.1, NC-030814.1, and NC-030818.1, among which NC-030810.1 had more up-regulated genes, and NC-030809.1 and NC-030813.1 had more down-regulated genes. As shown in Fig. 2f, differential TUCPs are primarily concentrated on chromosomes NC-030809.1, NC-030811.1, NC-030814.1, NC-030816.1, and NC-030812.1, among which NC-030809.1, NC-030811.1, NC-030814.1, and NC-030816.1 had more up-regulated genes and other chromosome differences. Although many genes were up-regulated, chromosome NC-030812.1 had more down-regulated genes than the others.

### Co-expressed and co-located genes of differential lncRNAs /TUCPs

LncRNAs have no encoding potential, thus the functions of these molecules are achieved by regulating related genes. The biological function of lncRNA was predicted by its location relationship (co-location) and expression correlation (co-expression). The threshold of co-location is set as lncRNAs 100 kb upstream and downstream. Among all 115 differential lncRNAs, 87 lncRNAs corresponded to 326 related genes by co-location analysis. A total of 114 lncRNAs corresponded to 2584 related genes by co-expression analysis.

The same method was used to predict the related genes of TUCP. Among the 22 differential TUCPs, 17 TUCPs corresponded to 73 related genes by co-location analysis, and 22 TUCPs corresponded to 904 related genes by co-expression analysis.

### The GO and KEGG analysis of the co-expression genes of differential lncRNAs

**Fig. 3:**
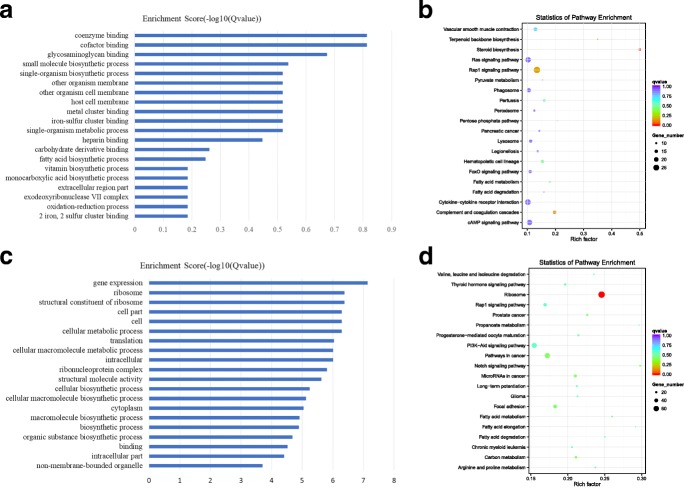
The GO and KEGG analysis of co-expressed genes of differential expressed lncRNAs. **a** The GO analysis of related gene of differential expressed and up-regulated lncRNAs, X-axis is qValue(−log10), Y-axis is the GO term. **b** KEGG pathway analyses of up-regulated lncRNAs. KEGG enrichment is measured by rich factor, qvalue and the number of genes enriched on this pathway. The larger the Rich factor, the greater the degree of enrichment. The value range of qvalue is [0,1]. The closer it is to zero, the more significant the enrichment is. Pathways with q ≤ 0.05 are defined as pathways that are significantly enriched in differentially expressed genes. **c** The GO analysis of related gene of down-regulated lncRNAs, X-axis is qValue(−log10), Y-axis is the GO term. **d** KEGG pathway analyses of down-regulated lncRNAs. KEGG enrichment is measured by rich factor, qvalue and the number of genes enriched on this pathway. The larger the Rich factor, the greater the degree of enrichment. The value range of qvalue is [0,1]. The closer it is to zero, the more significant the enrichment is. Pathways with q ≤ 0.05 are defined as pathways that are significantly enriched in differentially expressed genes

A total of 28 lncRNAs showed higher expression in luteal phase ovaries than in follicular phase ovaries. All of these molecules can be used for co-expressed analysis. These 28 lncRNAs corresponded to 1101 related genes by co-expression analyses. These related genes were used for the GO and KEGG enrichment analyses. The results of the GO enrichment analysis are shown in Fig. 3a and Additional file 2: Figure S2, and the terms cofactor binding and coenzyme binding were significantly enriched GO terms. Cofactors and coenzymes catalyze the synthesis of steroid hormones by enhancing enzyme activity. The KEGG enrichment analysis is shown in Fig. 3b. The most significant signal pathway is steroid biosynthesis (*P* = 5.16E-06). According to these data, the GO terms cofactor binding and coenzyme binding and the KEGG pathway of steroid biosynthesis have some common genes, such as SQLE, NSDH, HSD17B7, HRS1, and DHCR24, which were the most differentially expressed genes. These genes co-expressed with lncRNAs include XR_001919417.1, XR_001918177.1 and XR_001917326.1 (Additional file 6: Table S1). Therefore, in the luteal phase ovary, the highly expressed lncRNAs, such as XR_001919417.1, XR_001918177.1 and XR_001917326.1, were related to the synthesis of steroid hormones.

There were 87 highly expressed lncRNAs in the follicular phase ovary, and 86 of these molecules were used for co-expression analysis. These 86 lncRNAs corresponded to 1931 genes. These genes were used for the GO and KEGG enrichment analyses. The GO enrichment analysis is shown in Fig. 3c, and the terms gene expression (*p* = 1.62E-11), ribosome (*p* = 2.80E-10) and translation (*p* = 1.42E-09) were significantly enriched GO terms. The KEGG enrichment analysis is shown in Fig. 3d. The most significant signal pathway from the KEGG analysis is ribosome. According to these data, the GO terms gene expression, ribosome, translation and the KEGG pathway of ribosome share common genes. Many common genes, such as FAU, BCL2, and RPS6, were differentially expressed. The co-expressed lncRNAs include XR_001919841.1, XR_001917388.1 and XR_001918469.1 (Additional file 6: Table S1). Therefore, the highly expressed lncRNAs in the follicular phase ovary, such as XR_001919841.1, XR_001917388.1 and XR_001918469.1, were related to gene expression.

### The GO and KEGG analysis of co-located genes of differential lncRNAs

**Fig. 4:**
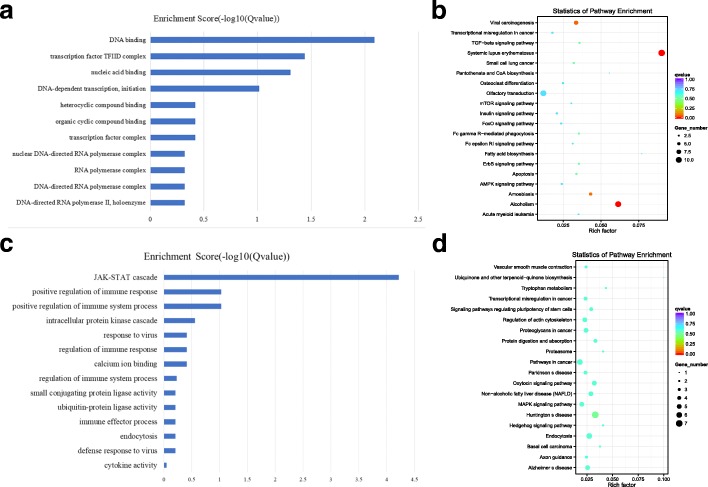
The GO and KEGG analysis of co-located genes of differential lncRNAs. **a** The GO analysis of related gene of differential expressed and up-regulated lncRNAs, X-axis is qValue(−log10), Y-axis is the GO term. **b** KEGG pathway analyses of up-regulated lncRNAs. KEGG enrichment is measured by rich factor, qvalue and the number of genes enriched on this pathway. The larger the Rich factor, the greater the degree of enrichment. The value range of qvalue is [0,1]. The closer it is to zero, the more significant the enrichment is. Pathways with q ≤ 0.05 are defined as pathways that are significantly enriched in differentially expressed genes. **c** The GO analysis of related gene of down-regulated lncRNAs, X-axis is qValue(−log10), Y-axis is the GO term. **d** KEGG pathway analyses of down-regulated lncRNAs. KEGG enrichment is measured by rich factor, qvalue and the number of genes enriched on this pathway. The larger the Rich factor, the greater the degree of enrichment. The value range of qvalue is [0,1]. The closer it is to zero, the more significant the enrichment is. Pathways with q ≤ 0.05 are defined as pathways that are significantly enriched in differentially expressed genes

A total of 28 lncRNAs showed higher expression levels in the luteal phase ovaries than those in the follicular phase ovaries, and 24 of these molecules could be used for co-location analysis. These 24 lncRNAs corresponded to 99 genes. These 99 genes were used for the GO and KEGG enrichment analyses. The GO enrichment analysis is shown in Fig. 4a and Additional file 3: Figure S3, and the term DNA binding was the most significantly enriched GO term. Many of these genes, such as PANK1, WRNIP1, and OTUD7B, were differentially expressed. The lncRNAs that co-localized with these genes were XR_001917326.1, LNC_002673, XR_309,871.3 and so on. (Additional file 6: Table S1). In summary, in the luteal phase ovary, the highly expressed lncRNAs, such as XR_001917326.1, LNC_002673, and XR_309,871.3, may be associated with the formation of the transcription initiation complex.

There were 87 lncRNAs with higher expression in the follicular phase ovaries, and 63 of these molecules could be used for co-location analysis. These 63 lncRNAs corresponded to 236 related genes. These 236 genes were used for the GO and KEGG enrichment analyses. The GO enrichment analysis is shown in Fig. 4c. JAK-STAT cascade (*p* = 1.36E-08) was the most significantly enriched GO term. STAT is a signal transcriptome and transcriptional activator that passes through the nucleus to regulate the expression of related genes when it is phosphorylated by JAK. According to the present results, many related genes of this term, such as LOC102182821, LOC108638069, LOC108638068, and LOC108638070, were differentially expressed genes. These related genes co-localized with XR_001296791.2 (Additional file 6: Table S1). In summary, in the follicle phase ovaries, the highly expressed lncRNA XR_001296791.2 was related to gene expression.

### The GO and KEGG analysis of co-expressed genes of differential TUCPs

**Fig. 5:**
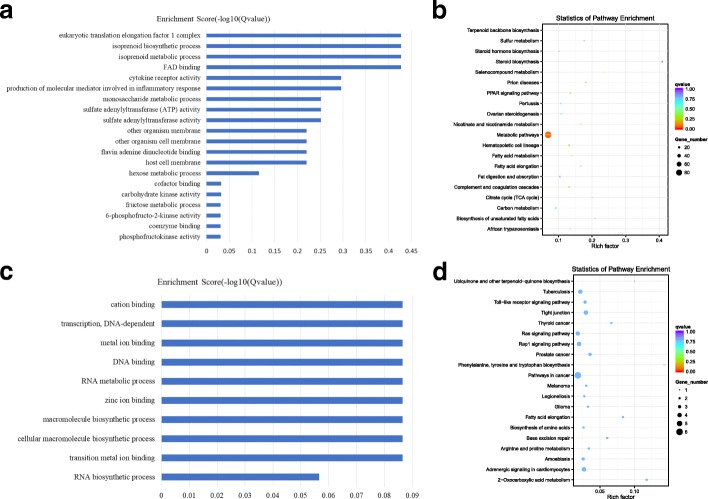
The GO and KEGG analysis of co-expressed genes of differential TUCPs. **a** The GO analysis of related gene of differential expressed and up-regulated TUCPs, X-axis is qValue(−log10), Y-axis is the GO term. **b** KEGG pathway analyses of up-regulated TUCPs. KEGG enrichment is measured by rich factor, qvalue and the number of genes enriched on this pathway. The larger the Rich factor, the greater the degree of enrichment. The value range of qvalue is [0,1]. The closer it is to zero, the more significant the enrichment is. Pathways with q ≤ 0.05 are defined as pathways that are significantly enriched in differentially expressed genes. **c** The GO analysis of related gene of down-regulated TUCPs, X-axis is qValue(−log10), Y-axis is the GO term. **d** KEGG pathway analyses of down-regulated TUCPs. KEGG enrichment is measured by rich factor, qvalue and the number of genes enriched on this pathway. The larger the Rich factor, the greater the degree of enrichment. The value range of qvalue is [0,1]. The closer it is to zero, the more significant the enrichment is. Pathways with q ≤ 0.05 are defined as pathways that are significantly enriched in differentially expressed genes

There were 16 TUCPs showing higher expression levels in the luteal phase ovaries than those in the follicular phase ovaries. All of these molecules could be used for co- expression analysis. These 16 TUCPs corresponded to 765 genes. These 765 genes were used for the GO and KEGG enrichment analyses. The GO enrichment analysis is shown in Fig. 5a and Additional file 4: Figure S4, and the terms isoprenoid metabolic process and isoprenoid biosynthetic process were significantly enriched GO terms. In these two terms, the related genes, such as FDPS, KDM1A, and AIFM1 were differentially expressed, and the TUCPs showing related genes co-expression include TUCP_001032, TUCP_000183, and TUCP_001362 (Additional file 6: Table S1). KEGG enrichment analysis is shown in Fig. 5b. The most significant signal pathway is steroid biosynthesis (6.73E-06). In the steroid biosynthesis signaling pathway, the related genes, such as EBP, DHCR24, and SQLE, were differentially expressed, and the TUCPs co-expressed with related genes include TUCP_001362, TUCP_001032, and TUCP_000183 (Additional file 6: Table S1). In summary, as the steroids belong to the isoprenoids, we conclude that in the luteal phase ovary, the highly expressed TUCPs, such as TUCP_001362, TUCP_001032 and TUCP_000183, were related to the synthesis of steroid hormones.

There were 6 TUCPs showing higher expression in the follicular phase ovary, and all of these molecules could be used for co-expression analysis. These 6 TUCPs corresponded to 157 related genes. These genes were used for the GO and KEGG enrichment analyses. The GO enrichment analysis is shown in Fig. 5c. Transcription, DNA-dependent, DNA binding and RNA biosynthetic process were significantly enriched GO terms. In the three terms, many related genes, such as AR, HMGB1 and CREM, were differentially expressed. The genes co-expressed with TUCPs are TUCP_000849, TUCP_000249, TUCP_001109 and so on (Additional file 6: Table S1). In summary, in the follicle phase ovaries, the highly expressed TUCPs, such as TUCP_000849, TUCP_000249 and TUCP_001109, were related to gene expression.

### The GO and KEGG analysis of co-located genes of differential TUCPs

**Fig. 6:**
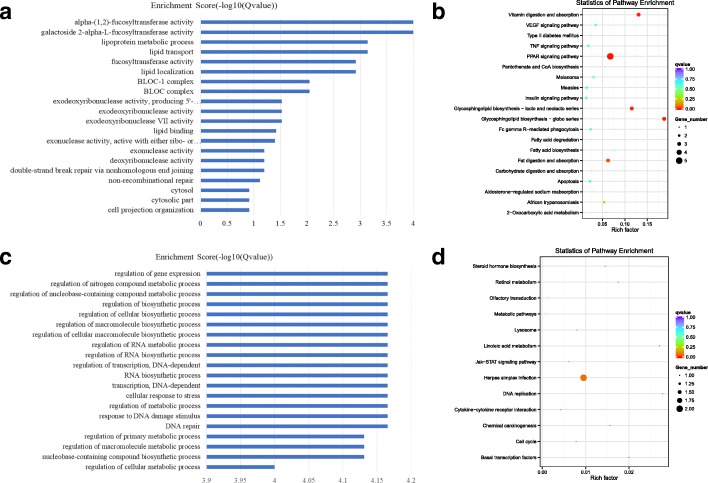
The GO and KEGG analysis of co-located genes of differential TUCPs. **a** The GO analysis of related gene of differential expressed and up-regulated TUCPs, X-axis is qValue(−log10), Y-axis is the GO term. **b** KEGG pathway analyses of up-regulated TUCPs. KEGG enrichment is measured by rich factor, qvalue and the number of genes enriched on this pathway. The larger the Rich factor, the greater the degree of enrichment. The value range of qvalue is [0,1]. The closer it is to zero, the more significant the enrichment is. Pathways with q ≤ 0.05 are defined as pathways that are significantly enriched in differentially expressed genes. **c** The GO analysis of related gene of down-regulated TUCPs, X-axis is qValue(−log10), Y-axis is the GO term. **d** KEGG pathway analyses of down-regulated TUCPs. KEGG enrichment is measured by rich factor, qvalue and the number of genes enriched on this pathway. The larger the Rich factor, the greater the degree of enrichment. The value range of qvalue is [0,1]. The closer it is to zero, the more significant the enrichment is. Pathways with q ≤ 0.05 are defined as pathways that are significantly enriched in differentially expressed genes

There were 16 TUCPs showing higher localization in the luteal phase ovaries than in the follicular phase ovaries, and 11 of these molecules could be used for co-location analysis. These 11 TUCPs corresponded to 45 genes. These 45 genes were used for the GO and KEGG enrichment analyses. The GO enrichment analysis is shown in Fig. 6a and Additional file 5: Figure S5. The terms lipoprotein metabolic process and lipid localization were significantly enriched GO terms. In these two terms, many related genes, such as APOA5, LOC102179867 and APOA1, were differentially expressed. These genes co-localized with TUCP_000751 TUCP_000882 (Additional file 6: Table S1). KEGG enrichment analysis is shown in Fig. 6b. The signal pathway of glycosphingolipid biosynthesis - globo series, glycosphingolipid biosynthesis - lacto and neolacto series was significantly enriched. In the two signal pathways, many related genes, such as LOC102178850, FUT1 and LOC102185028, were differentially expressed. These genes only co-localized with TUCP_000882. In summary, in the luteal phase ovary, the highly expressed TUCPs, TUCP_000751 and TUCP_000882, are related to the biosynthesis of lipid (Additional file 6: Table S1). The increase in lipid amount in the luteal phase ovary was beneficial to the synthesis of steroids.

There were 6 TUCPs showing higher localization in the follicular phase ovary, and all of these molecules could be used for co-location analysis. These 6 TUCPs corresponded to 28 genes. These 28 genes were used for the GO and KEGG enrichment analyses. The GO enrichment analysis is shown in Fig. 6c. The regulation of gene expression, regulation of RNA biosynthetic process, transcription and DNA-dependent were significantly enriched GO terms. In the three terms, many related genes, such as MCM7, TAF6 and ZSCAN2, were differentially expressed. The related genes co-localized with TUCP_000849, TUCP_000249, TUCP_001109 and so on. (Additional file 6: Table S1). The KEGG enrichment analysis results are shown in Fig. 6d, and the DNA replication signaling pathway was significantly enriched. The MCM7 gene of this pathway significantly differs, and this gene co-localized with TUCP_001109(Additional file 6: Table S1). In summary, in the follicle phase ovary, the highly expressed TUCPs, such as TUCP_000849, TUCP_000249, and TUCP_001109, were related to gene expression.

## Discussion

LncRNAs widely participate in mammalian processes; however, compared with humans and other mammals, there is limited knowledge of lncRNAs in goats [33–36], particularly how lncRNAs regulate the reproductive processes, such as ovulation and the lambing rate, in goats during ovarian development. In the present study, we identified and analyzed the lncRNAs and TUCPs in goat ovaries at the luteal phase and follicular phases by RNA-seq. To our knowledge, the present study is the first systematic study of the lncRNAs and TUCPs in the goat ovaries. The present report is also the first to identify differentially expressed lncRNAs and TUCPs in follicular and luteal ovaries.

In the present study, the goat ovary transcriptome of lncRNAs and TUCPs were provided. According to the base quality and composition analysis, the Q20 was 95.73–97.14%, and the GC content was 49.2–50.60%. These findings indicated that the library was successfully constructed and that the sequencing quality was good. According to the sequencing results, the average length of lncRNAs /TUCPs is shorter than that of mRNAs, and the number of exons is lower, the length of the open reading frame is shorter, and the expression level of lncRNAs /TUCPs are relatively lower than those of mRNA. These transcriptome features are consistent with those reported in previous studies [34, 36, 37]. Pearson’s correlation between the samples ranged from 0.861 to 0.935, which shows that the present study is reliable and that the sample selection is reasonable.

LncRNA usually do not encode protein and can achieve functions by binding to chromosome or protein, especially transcription factors [38]. Recent studies have shown that the biological functions of lncRNAs in goat ovaries can be predicted by studying relevant genes using co-expression and co-localization methods [39]. Many researches further confirmed the credibility of such predicted methods. For example, after predicted the related gene (*CDKN1A*) of lncRNA *PANDA* by co-expressed method, it was confirmed with some experiments that *PANDA* interacts with the transcription factor NF-YA to limit expression of related gene [40]. Therefore, the co-expression and co-localization genes of differential lncRNA/TUCP was analysis is as follows.

What are the main functions of lncRNAs and TUCPs that are highly expressed in the luteal phase ovary? According to the functional analysis of higher expressed lncRNA (co-expressing genes) in the luteal phase ovary, the terms coenzyme binding and cofactor binding are the most significant GO terms. Coenzymes and cofactors may catalyze the synthesis of steroid hormones by increasing enzyme activity. For example, the cytochrome P450c17 protein encoded by the CYP17A1 (all called cytochrome P450c17α) gene is a cytochrome P450 enzyme expressed in the ovary and testis. P450c17 has both 17α-hydroxylase and 17,20-lyase activities and is a typical membrane-bound bifunctional monooxygenase that plays a key role in the synthesis of steroid hormones [41] and plays an important role in maintaining the synthesis of sex hormones [42]. Therefore, the lncRNAs that are highly expressed in the luteal phase may be mainly related to the synthesis of steroid hormones. According to the functional analysis of more highly expressed TUCPs (co-expressing genes) in the luteal phase ovary, the term isoprenoid is the most significant GO term. Steroids are a general term for a large class of cyclopentadiene-perhydrophenanthrene derivatives widely distributed in the biosphere. These compounds belong to the group isoprenoids. According to the functional analysis of more highly expressed lncRNAs (co-location genes) in the luteal phase ovary, the term lipid synthesis was the most significant GO term. The formation and accumulation of lipid droplets in the luteal cells may be important for the production of steroids [43] . Ovarian function is associated with decreased lipid levels, particularly in the luteal phase [44] . Through the above functional analysis of co-expressed and co-localized genes of TUCPs, we propose that TUCPs and lncRNAs highly expressed in the luteal phase ovary may be related to the synthesis of steroid hormone. In summary, the present results indicate that the high expression of lncRNA and TUCP in the luteal phase ovary may be mainly related to the synthesis of steroid hormones. We screened for lncRNAs/TUCPs that may be involved in steroid hormone synthesis, such as XR_001919417.1 and so on (Additional file 6: Table S1). Steroid hormones mainly include estrogen and progesterone, which are secreted by the gonads. These molecules can regulate the ovarian function in goats and other mammals. Consistent with Lee et al., knocking out the EGR-1 gene causes sterility in mice [45]. During pregnancy, estrogen plays a role in the initiation of labor and is conducive to childbirth. Progestogen can promote embryo implantation and maintain pregnancy. The secretion of estrogen and progesterone has an important influence on the reproductive function of animals [7]. In the luteal phase of the goat ovary, the follicle ruptures and forms a corpus luteum after ovulation. The corpus luteum mainly secretes a large amount of progesterone. Therefore, we propose that the difference between the ovary during the luteal phase and the follicular phase is mainly related to the synthesis of progesterone in steroid hormones. The above results can provide evidence that lncRNAs /TUCPs regulate the synthesis of progesterone in the luteal phase and influence the reproductive performance of animals.

What are the major functions of lncRNA and TUCP that are highly expressed in the follicular phase ovarian? According to the functional analysis of more highly expressed lncRNAs (co-expressing genes) in the follicular phase ovary, the terms ribosomes and translation were the most significant GO terms. The ribosome is the site of protein biosynthesis resulting from translation of messenger RNA (mRNA). The genetic information stored in the DNA sequence is transcribed and translated into biologically active protein molecules that enable organisms to exhibit corresponding traits. These actions are the most important process of gene expression. According to the functional analysis of more highly expressed TUCPs (both co-expressed and co-localized genes) in the follicular phase ovary, the terms transcription, DNA-dependent, and RNA biosynthetic processes are the common GO terms, with significant differences. Transcription is the first stage of gene expression and the main stage of gene regulation. Gene expression consists of transcription and translation processes. In summary, analyses with co-expression or co-location methods show that highly expressed lncRNAs /TUCPs in the follicular phase ovary are mainly associated with gene expression. We screened lncRNAs /TUCPs related to gene expression, such as XR_001296791.2 and so on (Additional file 6: Table S1). In the follicular phase goat ovaries, the follicles continue developing, and the oocytes continue growing and achieve maturation. An increasing number of maternal factors accumulate during oocyte growth and development [46] . This process requires large amounts of gene expression [47, 48]. Studies have shown that gene expression is an indispensable step in the oogenesis and maturation of oocyte [48–50]. Therefore, we propose that the difference between the ovarian follicular phase and the luteal phase is mainly related to oogenesis and oocyte maturation. One of the main functions of lncRNAs is to regulate gene expression and participate in various physiological and developmental processes of organisms [51–53] . The above results can provide research ideas for how these lncRNAs /TUCPs regulate gene expression and affect the growth and maturation of oocytes, further affecting animal reproductive performance.

Some new lncRNAs were discovered to both co-located and co-expressed to a same genes. Three pairs of them were higher expressed in follicular stage ovarian: XR-001917610.1 and STAG2; XR-001919591.1 and ST6GALNAC1; XR-001919196.1 and LOC102180063. Two pairs of them were higher expressed in luteal stage ovarian: XR-309871.3 and BOLA1; XR-001919152.1 and LOC108633292. Further research these lncRNAs maybe help to understand the regulation of reproduction.

## Conclusion

In the present study, we conducted genome-wide RNA-seq for follicular and luteal ovaries in goat and provided the transcriptome profile of the lncRNAs and TUCPs in the goat ovaries. In addition, we screened the lncRNAs /TUCPs associated with hormone secretion and follicular development. The present study also provided fundamental data for studying the regulation mechanisms of lncRNAs /TUCPs in goat reproduction.

## Additional files

Additional file 1: **Figure S1.** The comparison of features of genomic characteristics between lncRNA/TUCP and mRNA. A. The distribution of exon number in the lncRNAs and mRNAs. B. The distribution of average length in the lncRNAs and mRNAs. C. The distribution of ORF in the lncRNAs and mRNAs. D. The distribution of exon number in the TUCPs and mRNAs. E. The distribution of average length in the TUCPs and mRNAs. F. The distribution of ORF in the TUCPs and mRNAs. (PDF 860 kb)

Additional file 2: **Figure S2.** The Directed Acyclic Graph (DAG) of GO analysis of co-expressed genes of differential lncRNAs. DAG is a graphical display of GO gene enrichment analysis results for differential lncRNAs. Branches represent containment relationships, and the functional scope defined from top to bottom is getting smaller and smaller. The depth of color represents the degree of enrichment. We have plotted the DAG maps of biological processes, cellular components and molecular functions separately. A. The DAG of biological process. B. The DAG of cellular component. C. The DAG of molecular function. (PDF 446 kb)

Additional file 3: **Figure S3.** The Directed Acyclic Graph (DAG) of GO analysis of co-located genes of differential lncRNAs. A. The DAG of biological process. B. The DAG of cellular component. C. The DAG of molecular function (PDF 478 kb)

Additional file 4: **Figure S4.** The Directed Acyclic Graph (DAG) of GO analysis of co-expressed genes of differential TUCPs. A. The DAG of biological process. B. The DAG of cellular component. C. The DAG of molecular function (PDF 572 kb)

Additional file 5: **Figure S5.** The Directed Acyclic Graph (DAG) of GO analysis of co-located genes of differential TUCPs. A. The DAG of biological process. B. The DAG of cellular component. C. The DAG of molecular function. (PDF 440 kb)

Additional file 6: **Table S1.** The list of reproductive lncRNA, TUCP and related genes. (XLSX 15 kb)

## Abbreviations

AIFM1: Apoptosis inducing factor mitochondria associated 1
APOA1: Apolipoprotein A1
AR: Androgen receptor
BCL2: Apoptosis regulator
CNCI: Coding-Non-Coding Index
CPC: Coding Potential Calculator
CREM: CAMP responsive element modulator
DHCR24: 24-dehydrocholesterol reductase
EBP: Emopamil binding protein (sterol isomerase)
FAU: Ubiquitin like and ribosomal protein S30 fusion
FDPS: Farnesyl diphosphate synthase
FPKM: Expected number of Fragments Per Kilobase of transcript sequence per Millions base pairs sequenced
FUT1: Fucosyltransferase 1 (H blood group)
GO: Gene Ontology
HMGB1: High mobility group box 1
HSD17B7: Ydroxysteroid 17-beta dehydrogenase 7
KDM1A: Lysine demethylase 1A
KEGG: Kyoto Encyclopedia of Genes and Genomes
lncRNAs: Long non-coding RNAs
LOC102178850: Galactoside 2-alpha-L-fucosyltransferase 2-like
LOC102179867: Apolipoprotein A-I
LOC102182821: Interferon lambda-3
LOC102185028: Galactoside 2-alpha-L-fucosyltransferase 2
LOC108638068: Interferon lambda-4-like
LOC108638069: Interferon lambda-4-like
LOC108638070: Interferon lambda-3-like
MAML1: Mastermind like transcriptional coactivator 1
MCM7: Minichromosome maintenance complex component 7
OTUD7B: OTU deubiquitinase 7B
PANK1: Pantothenate kinase 1
PFAM: Pfamscan
PLIN2: Perilipin 2
RPS6: Ribosomal protein S6
SQLE: Squalene epoxidase
SQLE: Squalene epoxidase
TAF6: TATA-box binding protein associated factor 6
TUCPs: Transcripts of uncertain coding potential
WRNIP1: Werner helicase interacting protein 1
ZSCAN2: Zinc finger and SCAN domain containing 2
