# Supplementary figures and images for: Filtered reproductive long non-coding RNAs by genome-wide analyses of goat ovary at different estrus periods
Source: BMC Genomics. 2018 Dec 4;19:866. doi: 10.1186/s12864-018-5268-7 (PMC6278114; doi:10.1186/s12864-018-5268-7)

**a**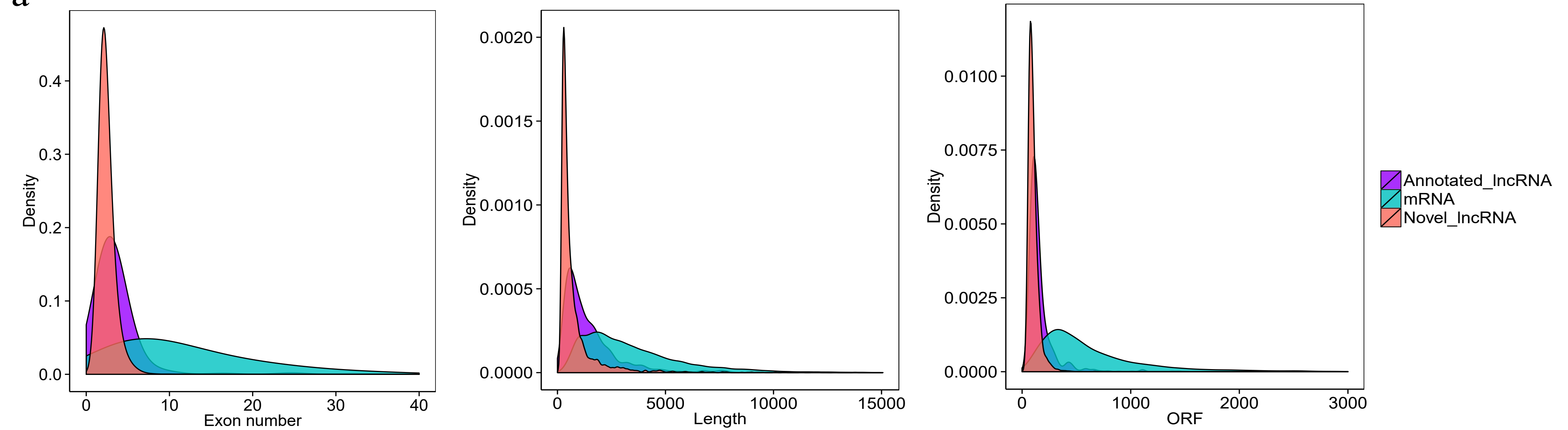**b**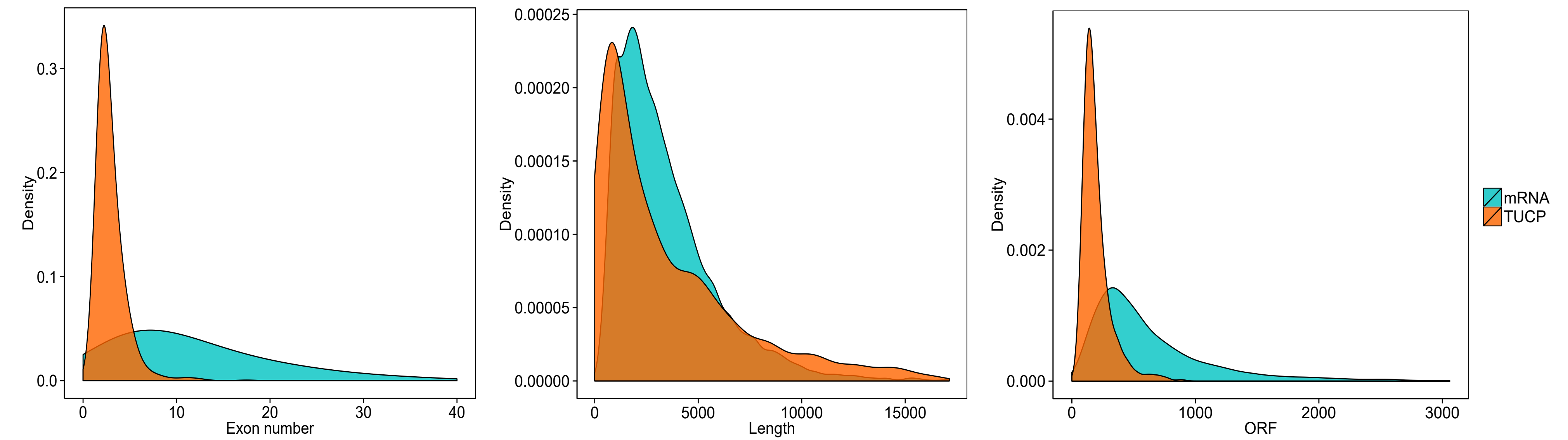

Supplement: Supplementary file 1 — Figure S1. The comparison of features of genomic characteristics between lncRNA/TUCP and mRNA. A. The distribution of exon number in the lncRNAs and mRNAs. B. The distribution of average length in the lncRNAs and mRNAs. C. The distribution of ORF in the lncRNAs and mRNAs. D. The distribution of exon number in the TUCPs and mRNAs. E. The distribution of average length in the TUCPs and mRNAs. F. The distribution of ORF in the TUCPs and mRNAs. (PDF 860 kb) [file 12864_2018_5268_MOESM1_ESM.pdf]

a

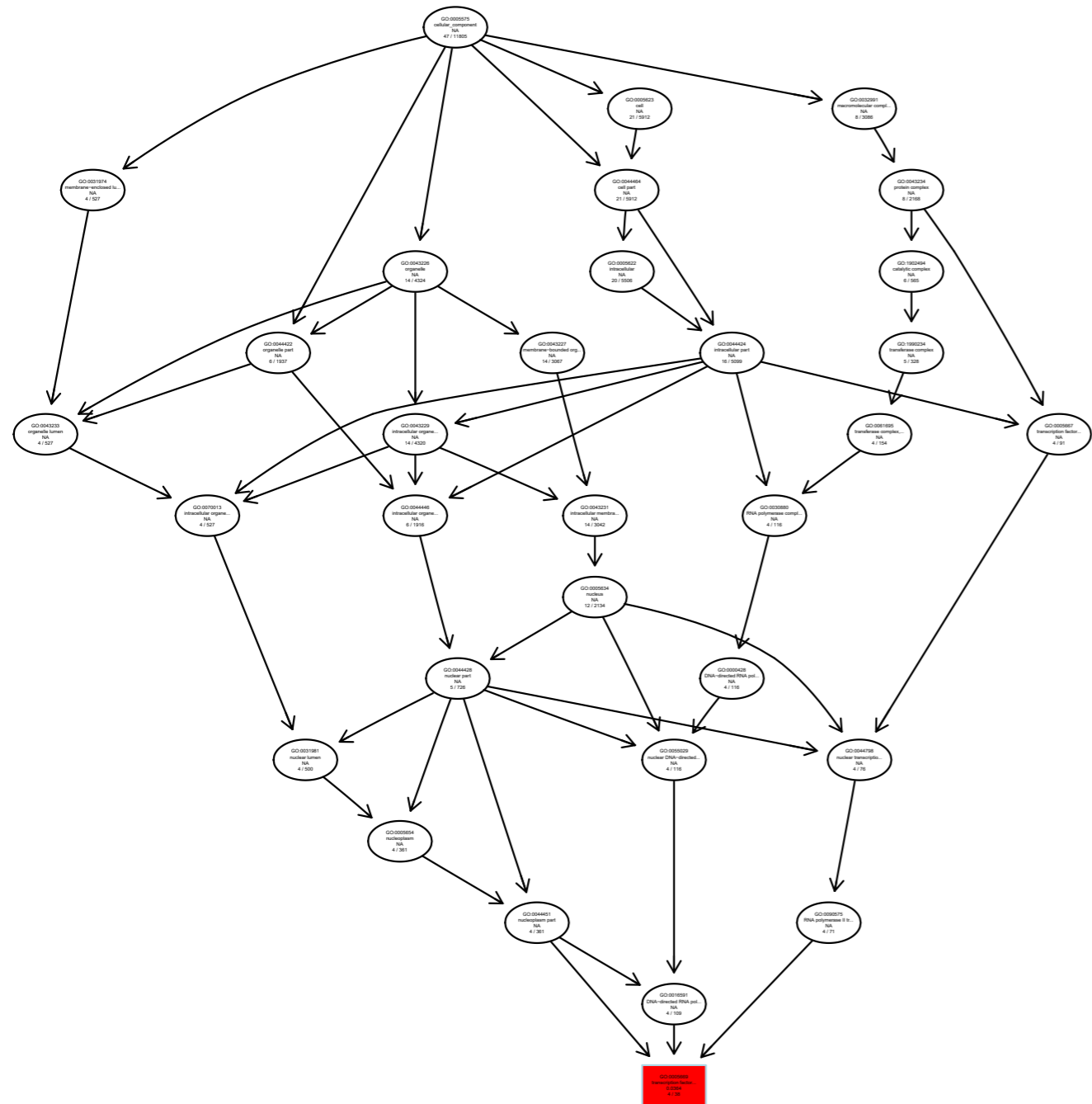

b

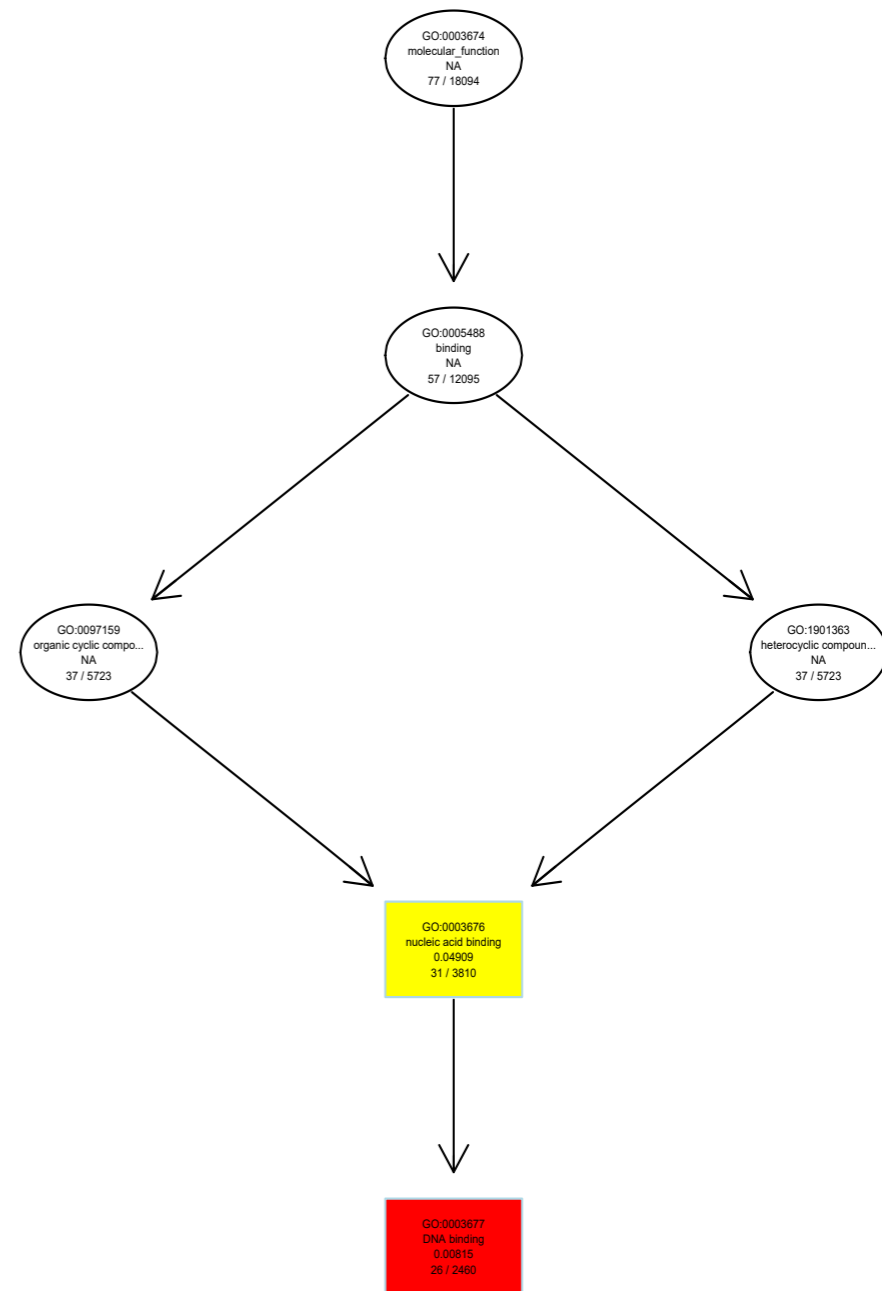

Supplement: Supplementary file 2 — Figure S2. The Directed Acyclic Graph (DAG) of GO analysis of co-expressed genes of differential lncRNAs. DAG is a graphical display of GO gene enrichment analysis results for differential lncRNAs. Branches represent containment relationships, and the functional scope defined from top to bottom is getting smaller and smaller. The depth of color represents the degree of enrichment. We have plotted the DAG maps of biological processes, cellular components and molecular functions separately. A. The DAG of biological process. B. The DAG of cellular component. C. The DAG of molecular function. (PDF 446 kb) [file 12864_2018_5268_MOESM2_ESM.pdf]

a

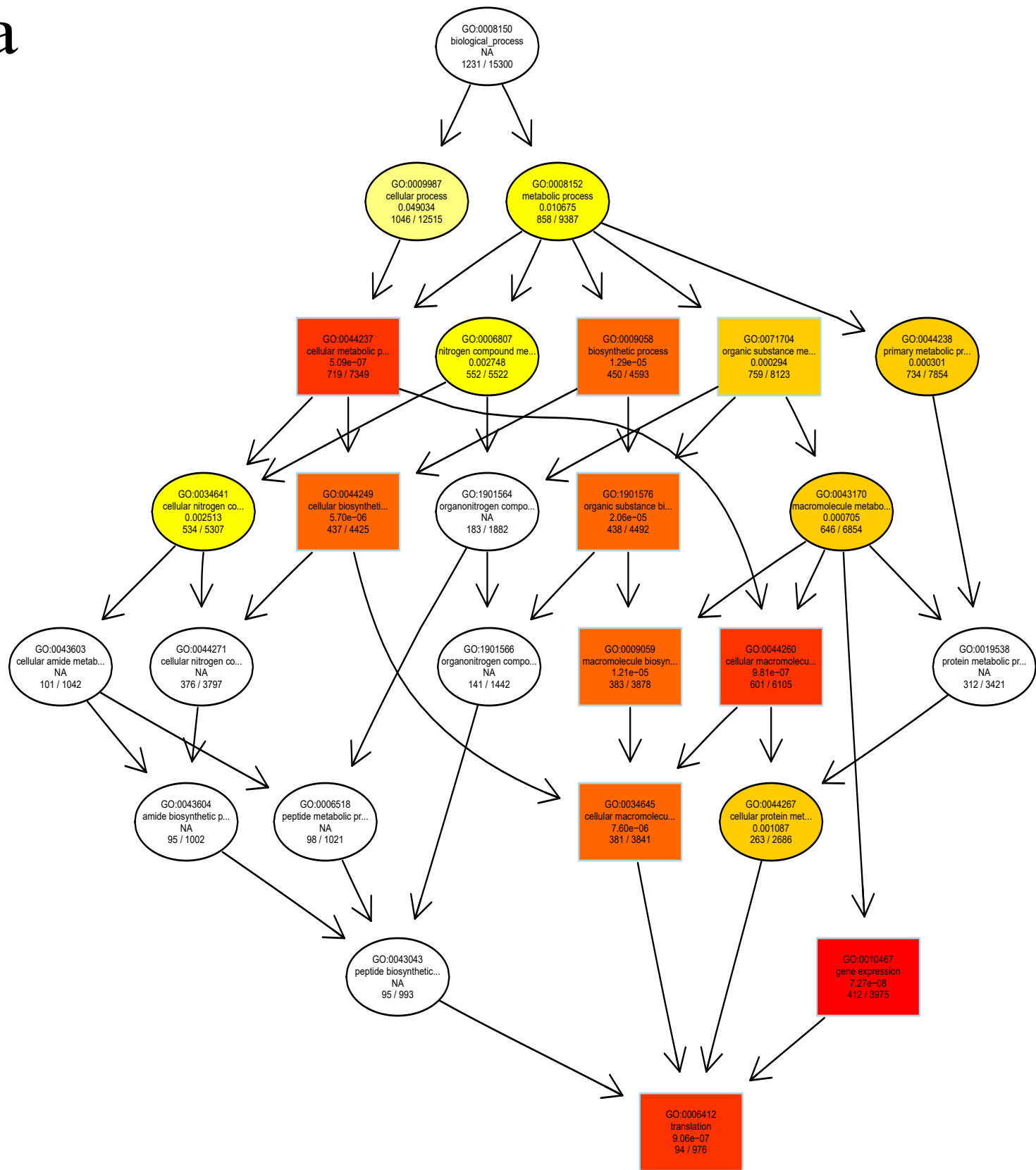

b

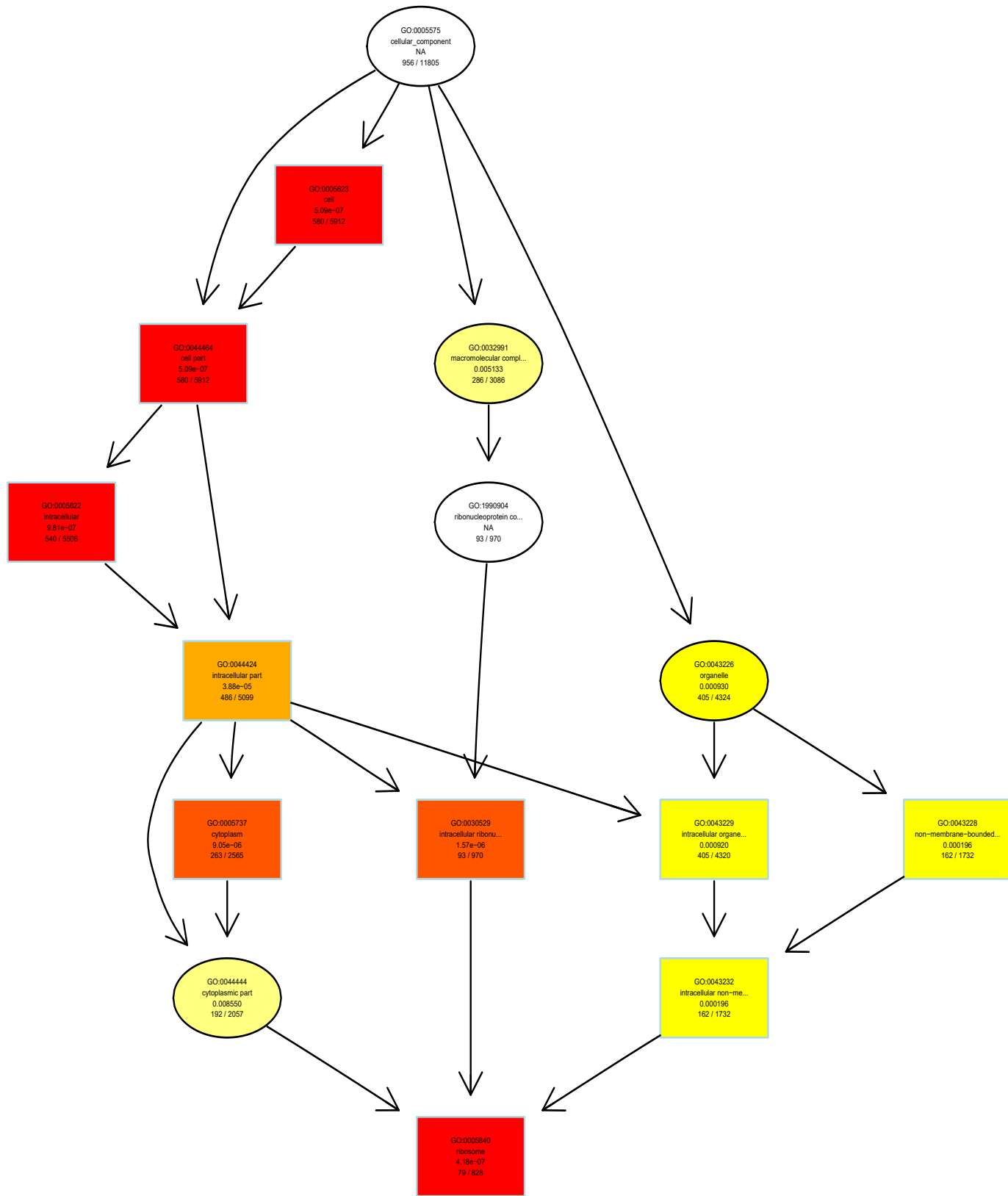

c

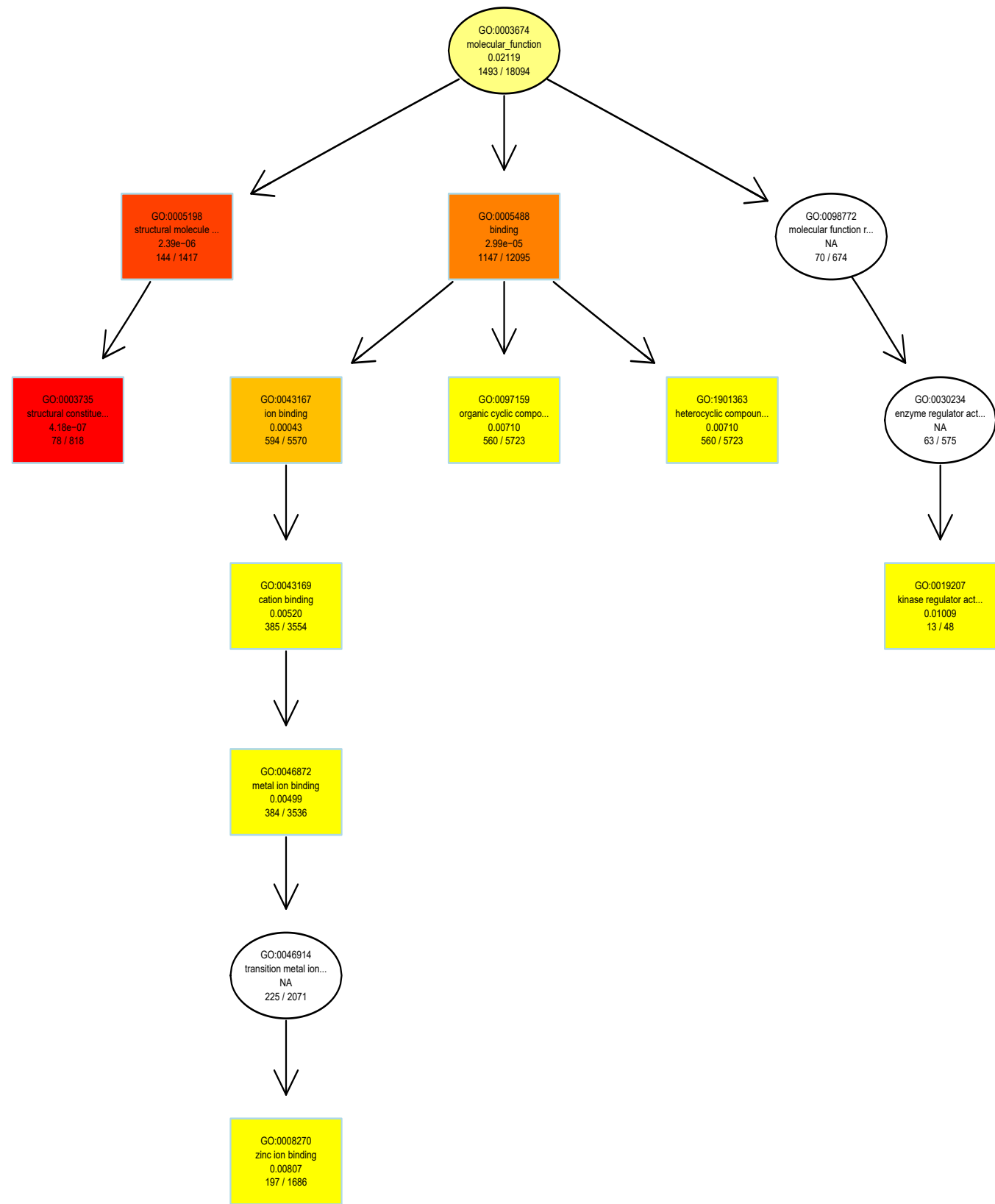

Supplement: Supplementary file 3 — Figure S3. The Directed Acyclic Graph (DAG) of GO analysis of co-located genes of differential lncRNAs. A. The DAG of biological process. B. The DAG of cellular component. C. The DAG of molecular function (PDF 478 kb) [file 12864_2018_5268_MOESM3_ESM.pdf]

a

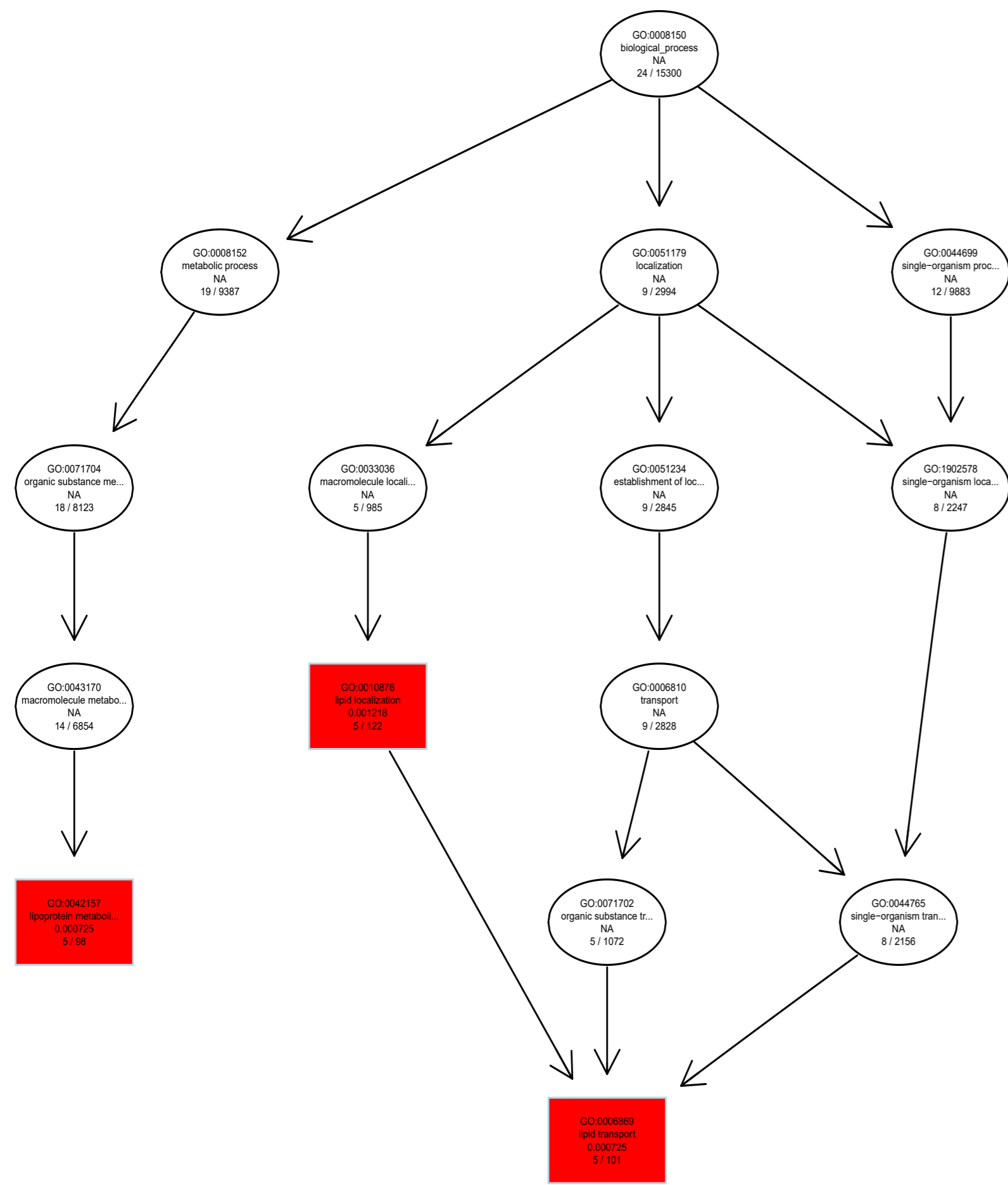

b

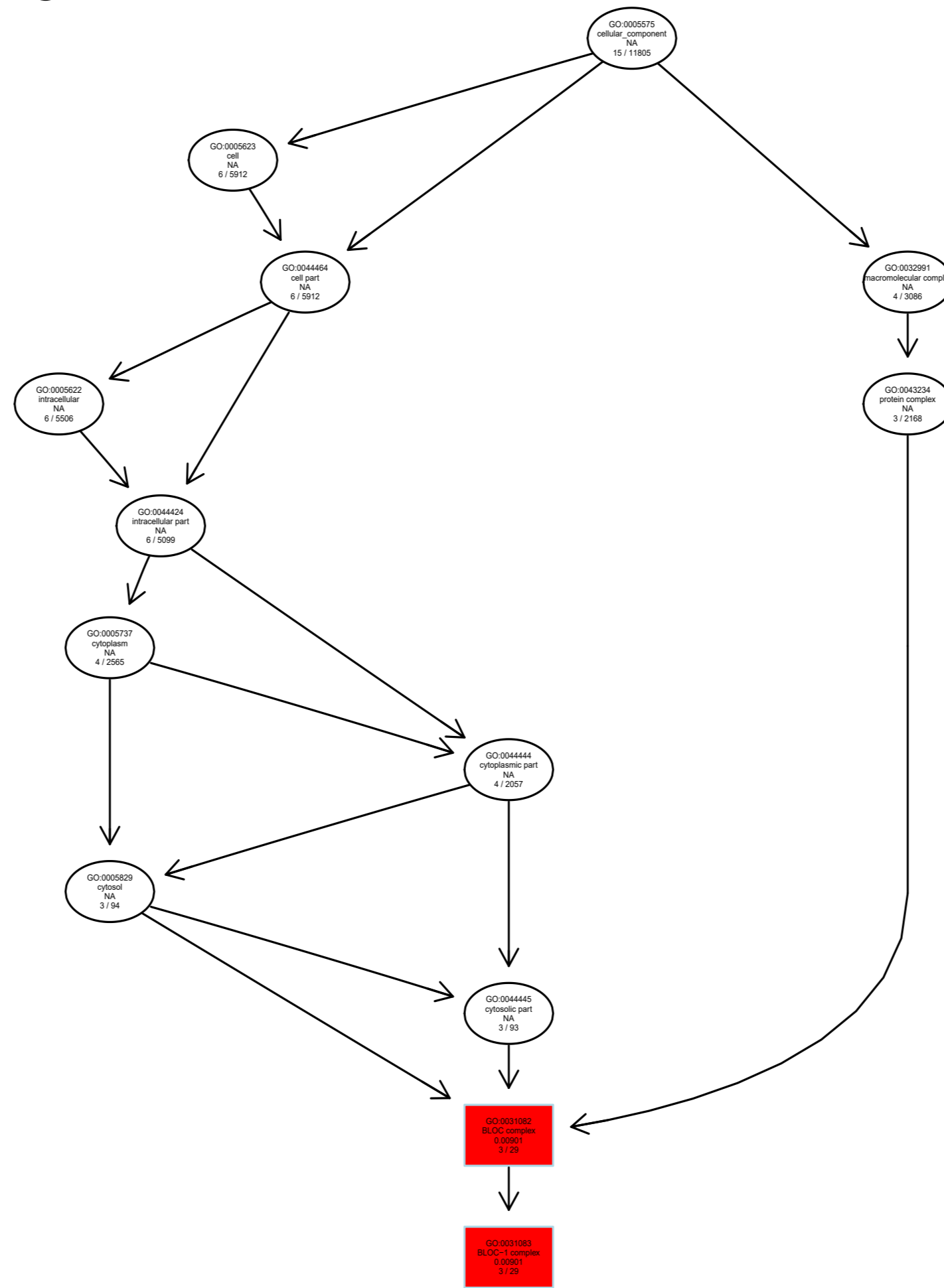

c

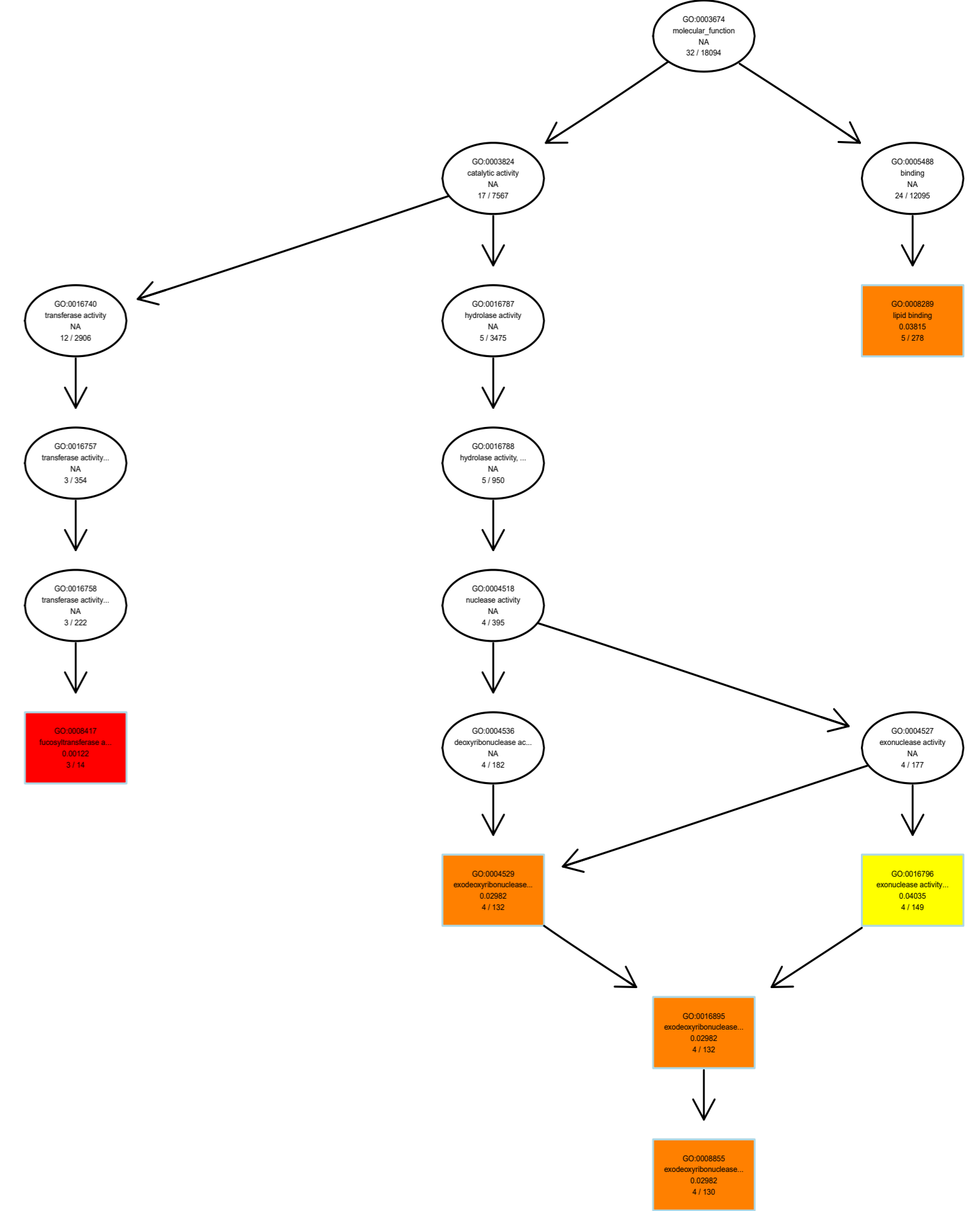

d

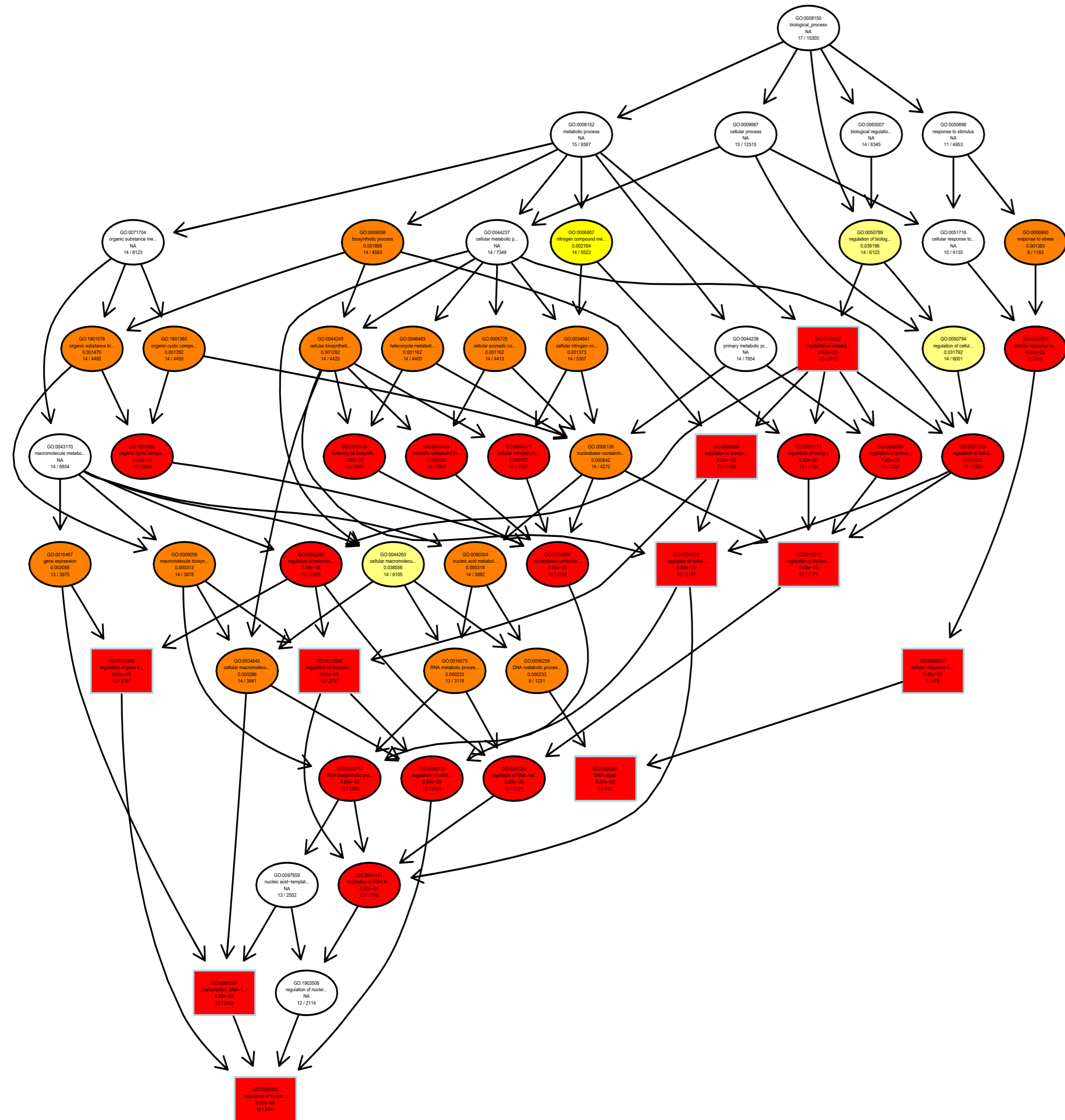

e

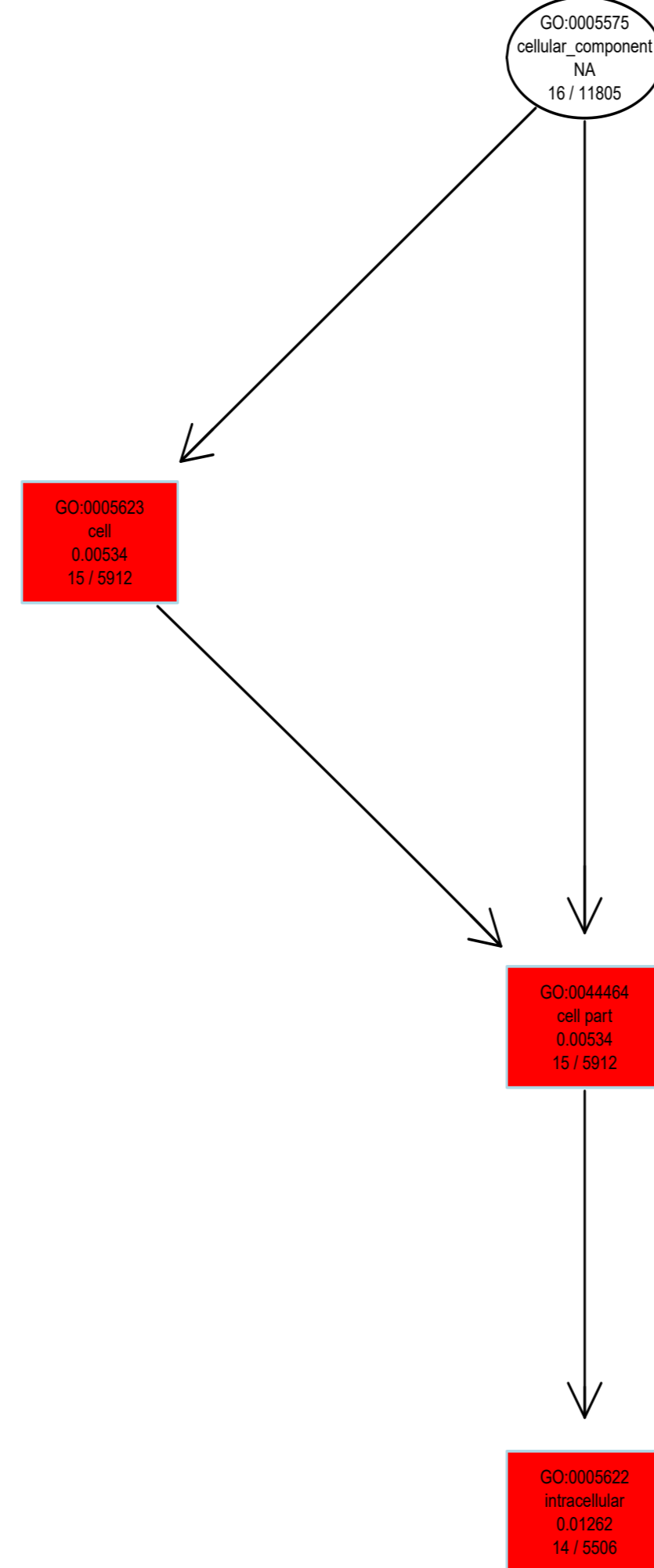

f

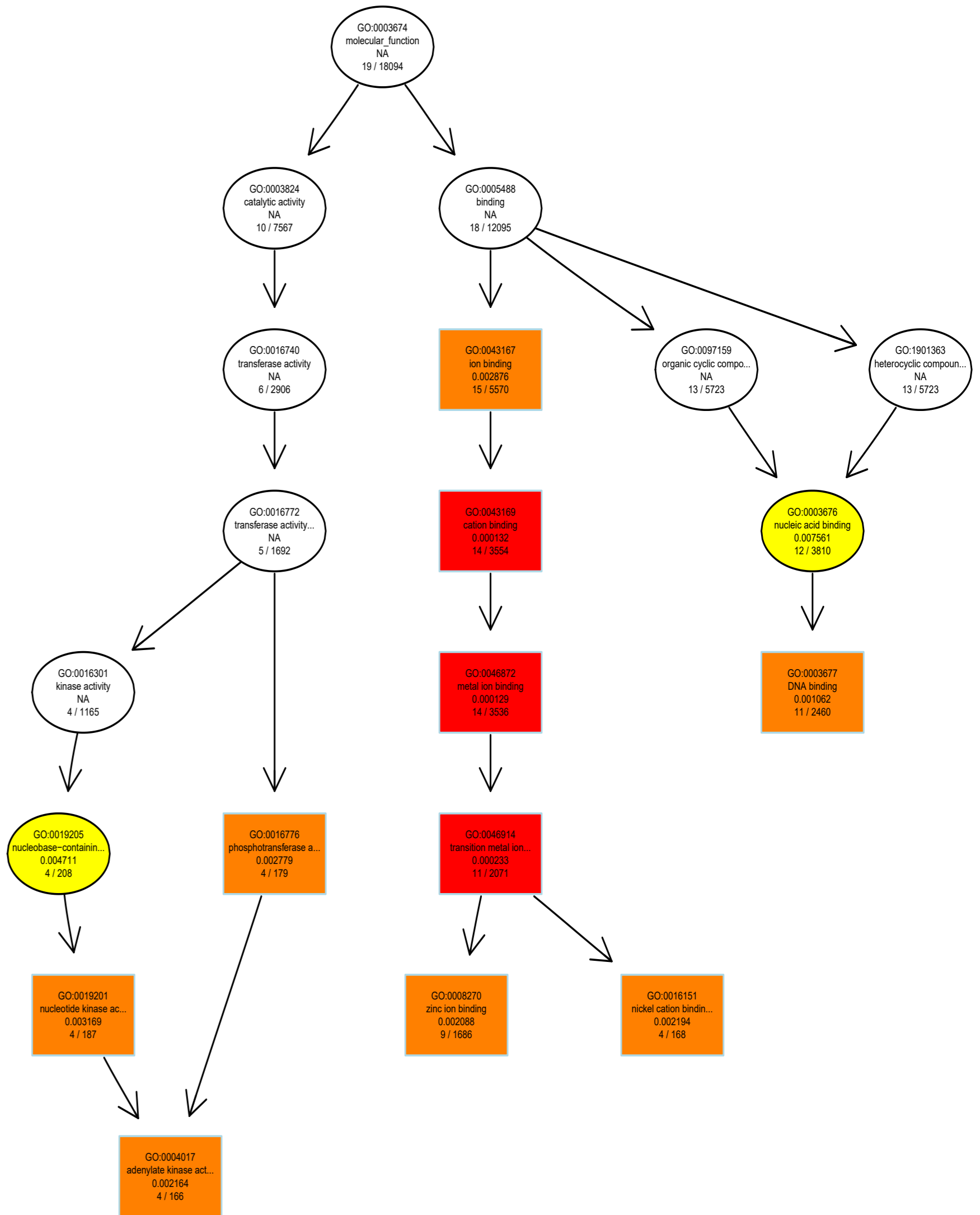

Supplement: Supplementary file 4 — Figure S4. The Directed Acyclic Graph (DAG) of GO analysis of co-expressed genes of differential TUCPs. A. The DAG of biological process. B. The DAG of cellular component. C. The DAG of molecular function (PDF 572 kb) [file 12864_2018_5268_MOESM4_ESM.pdf]

a

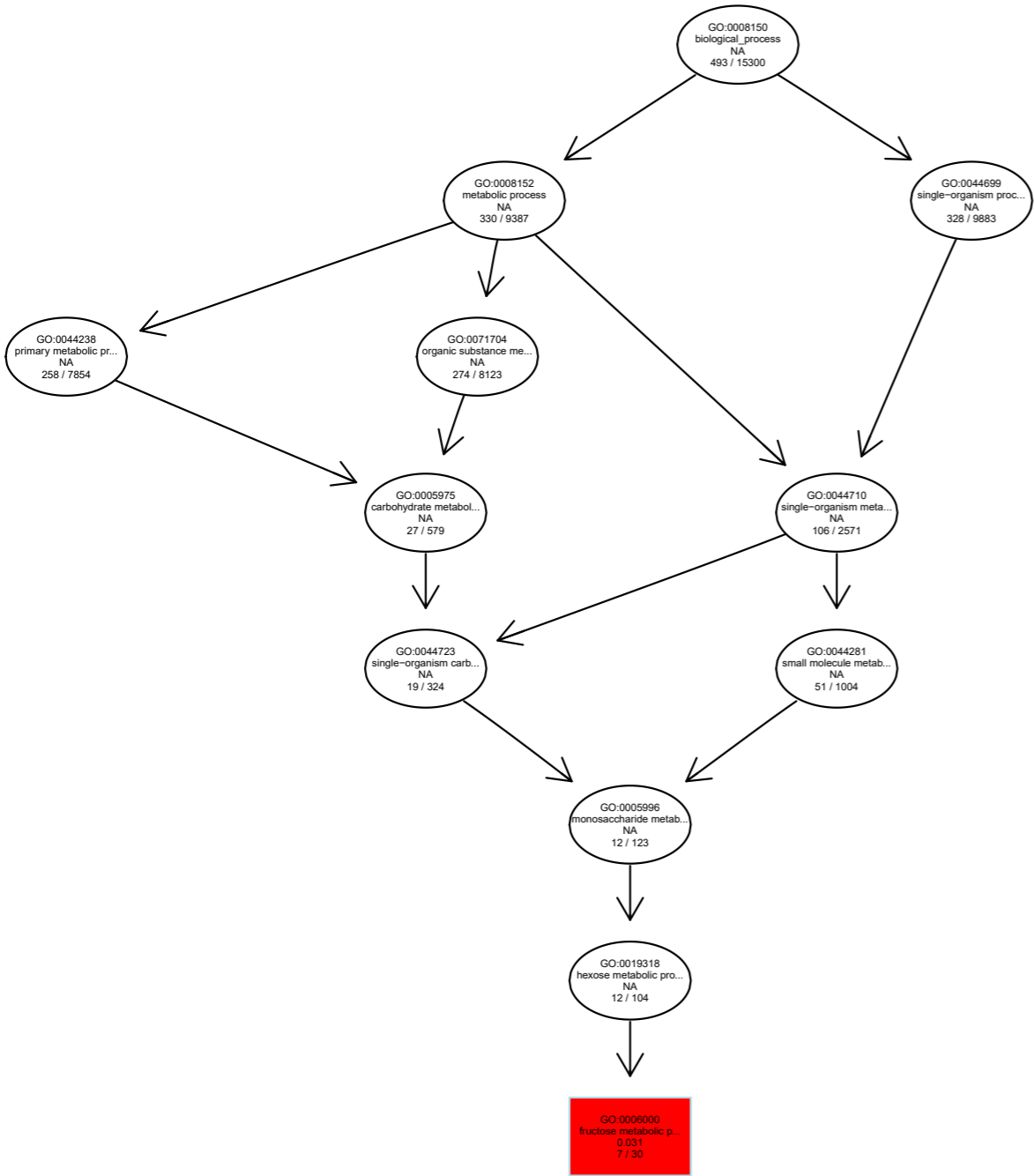

b

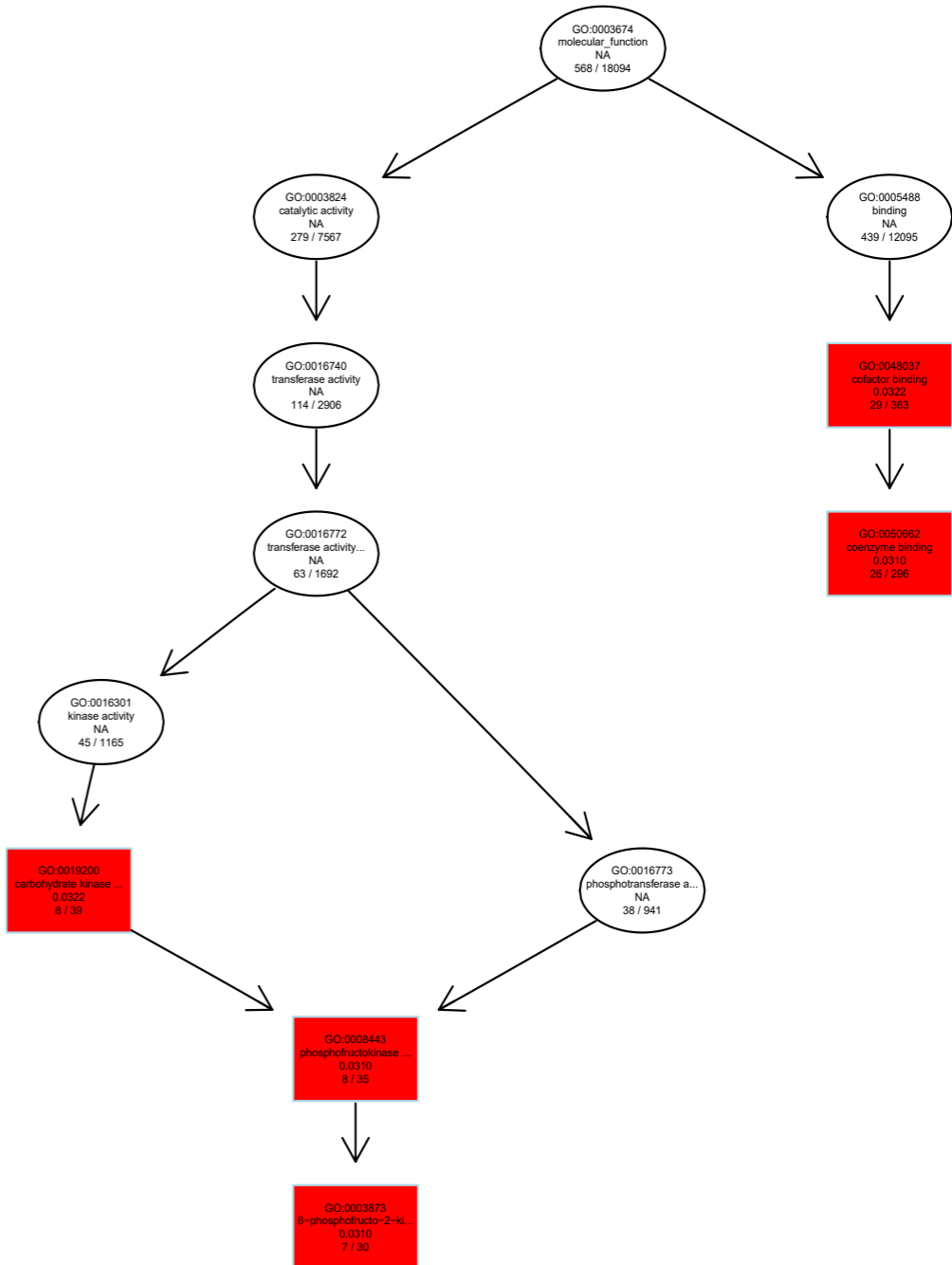

Supplement: Supplementary file 5 — Figure S5. The Directed Acyclic Graph (DAG) of GO analysis of co-located genes of differential TUCPs. A. The DAG of biological process. B. The DAG of cellular component. C. The DAG of molecular function. (PDF 440 kb) [file 12864_2018_5268_MOESM5_ESM.pdf]
